# Prevalence of methicillin-resistant *Staphylococcus aureus* and associated factors from surgical ward inpatients at Shaafi Hospital, Mogadishu, Somalia

**DOI:** 10.1016/j.ijregi.2025.100717

**Published:** 2025-07-30

**Authors:** Shafie Abdulkadir Hassan, Mowlid Abdikarin Mohamed, Zeynab Abdi Salad, Nimo Dahir Mohamed, Fardowsa Abdulahi Ahmed, Farhan Abdiaziz Dirie, Mohamed Khalif Abdulle, Abdifetah Ibrahim Omar, Nur Rashid Ahmed

**Affiliations:** 1 Department of Medical Laboratory Sciences, Faculty of Medicine and Health Sciences, Jamhuriya University of Science and Technology, Mogadishu, Somalia; 2 Department of Medicine and Surgery, Faculty of Medicine and Health Sciences, Jamhuriya University of Science and Technology, Mogadishu, Somalia; 3 Institute of Advanced Research, Jamhuriya University of Science and Technology, Jamhuriya University of Science and Technology, Mogadishu, Somalia

**Keywords:** MRSA, Prevalence, Surgical wounds, Risk factors, Antimicrobial susceptibility

## Abstract

**Objectives:**

Methicillin-resistant *Staphylococcus aureus* (MRSA) causes severe infections with high mortality rates and health care costs. This study aimed to evaluate the prevalence of MRSA and its associated factors among patients with wound infections at Shaafi Hospital in Mogadishu, Somalia.

**Methods:**

Wound swabs were collected from 418 patients with surgical wounds and cultured on Mannitol salt agar. *S. aureus* was identified using conventional methods, and antimicrobial susceptibility was performed using the disk diffusion method. Data analysis was conducted using SPSS software, with results presented as frequencies and percentages. Chi-square tests and binary logistic regression were performed to identify associations between MRSA and independent variables.

**Results:**

*S. aureus* was isolated in 116 (27.8%) patients, with 31 (26.7%) cases identified as MRSA. The prevalence of MRSA was higher among females (10, 27%) than males (21, 26.6%). The most frequent MRSA was observed in the 21-30 years age group (13, 24.5%). Patients with deep wounds had a significant association with MRSA (adjusted odds ratio [AOR]: 4.32, 95% confidence interval [CI]: 1.042-17.92, *P* <0.044). Hospital stay of longer than 72 hours (AOR: 4.1, 95% CI: 1.22-13.80, *P* <0.022) and history of antibiotic use (AOR: 1.12, 95% CI: 0.17-7.15, *P* <0.018) were also found significant risk factors. Penicillin was 100% resistant to MRSA, followed by 58% to cotrimoxazole, 54.8% to gentamycin, and 51.6% to ciprofloxacin, whereas MRSA was 100% susceptible to vancomycin.

**Conclusions:**

The prevalence of MRSA among inpatients with surgical wounds is considerable, with specific risk factors such as deep wounds, prolonged hospital stays, and previous antibiotic use significantly increasing the likelihood of MRSA infection. Antibiogram results indicated a significant resistance to frequently used antibiotics. This emphasizes the importance of implementing effective infection control measures and practicing antibiotic stewardship to manage MRSA in hospitals.

## Highlights

• Methicillin-resistant *Staphylococcus aureus* prevalence was 26.7% among surgical wound patients in a Shaafi Hospital.
• Deep wounds, long hospital stays, and previous antibiotic use increased methicillin-resistant *S. aureus* risk.
• High resistance was observed to common antibiotics.
• There is an urgent need for infection control and stewardship in Shaafi Hospital.

## Introduction

*Staphylococcus aureus* is a life-threatening bacterium responsible for invasive diseases, with the methicillin-resistant *S. aureus* (MRSA) strain an important cause of death worldwide [1]. MRSA refers to *S. aureus*, which has developed resistance to methicillin due to the production of β-lactamase enzymes and acquisition of the mecA gene [[2], [3], [4]]. It affects healthy and people with weakened immune systems but is particularly prevalent in hospital settings, contributing significantly to health care–associated infections [5]. MRSA is also found in the community, spreading through skin-to-skin contact, sharing of personal items, or being in crowded environments [6]. The primary factor behind MRSA’s antibiotic resistance is its ability to alter its genetic makeup, rendering many drugs ineffective [7]. This resistance raises concerns because it limits treatment options and increases the risk of complications [8]. Infections caused by MRSA often necessitate the use of stronger antibiotics, leading to increased side effects, prolonged hospital stays, and elevated health care costs [9]. Preventing MRSA requires proper hand hygiene, effective wound cleaning, and adherence to infection control protocols in hospitals and health care facilities [10]. Early identification and isolation of MRSA cases are vital for limiting its spread, especially in health care settings [11]. In 2019, antimicrobial resistance caused over 100,000 global deaths, with MRSA significantly contributing due to higher morbidity and mortality rates than Methicillin Susceptible Staphylococcus Aureus (MSSA) [12,13]. Somalia has a notable gap in research data on antimicrobial resistance, including MRSA, highlighting a critical gap. Somalia, a country recovering from decades of civil war, faces significant challenges in combating antimicrobial resistance, especially MRSA, due to fragile health infrastructure, limited laboratory facilities, and the fact that only a few laboratories can perform culture and susceptibility testing, which leads to inadequate diagnosis and management of infections [14]. Therefore, this study focused on assessing the prevalence of MRSA and the factors associated with Surgical Ward inpatients at Shaafi Hospital, Mogadishu, Somalia. The results of this study influence public health, increase infection prevention, improve patient care, and direct future research and policy in Somalia’s health care system.

## Methods

### Study design

This hospital-based correctional study was conducted at Shaafi Hospital, Mogadishu, Somalia from December 2023 to June 2024 and focused on inpatients with surgical wards.

### Selection criteria

#### Inclusion criteria

All participants with infected wounds were part of the study.

#### Exclusion criteria

Patients with clean wounds lacking pus or those severely ill were not part of the study.

### Study variables

The dependent variable in this study was the occurrence of MRSA*,* which represents the outcome. The independent variables included sex, age, wound type, site of infection, hospital stay duration, wound depth, level of contamination, operation type and duration, occupation, smoking habits, antibiotic use, recent traditional medical treatments, and hospitalization history.

### Sample size determination

A sample size of 422 was calculated using a single proportion formula, based on an assumed prevalence of 19.6% from a previous Ethiopian study and a 5% margin of error [15]. After excluding four incomplete cases, 418 participants were included.

### Sample collection and processing

Levine’s technique was used to collect wound samples after cleaning the area with sterile gauze soaked in 70% alcohol. To enhance bacterial recovery, double swabs were collected at different times. Although Levine’s technique allows semi-quantitative assessment of microbial load, in this study, it was applied qualitatively due to limited laboratory resources and the absence of equipment for quantitative analysis. All swabs were transported from Shaafi Hospital to the Jamahiriya Research Laboratory within 30-60 minutes in sterile containers placed in a portable cool box maintained at 4-8°C to ensure specimen integrity. After being transported, the samples were cultivated to mannitol salt agar and incubated at 37°C. *S. aureus* was subsequently isolated and identified through Gram staining, catalase testing, and coagulase testing [16]. Isolates were preserved in 20% glycerol broth at −80°C and sub-cultured periodically on nutrient agar to ensure viability and purity.

### Detection of MRSA and antimicrobial susceptibility testing

Antibiotic susceptibility testing was performed using the disk diffusion method in accordance with Clinical and Laboratory Standards Institute guidelines (2020). However, due to resource limitations, the D-zone test for detecting inducible clindamycin resistance was not performed [17]. A bacterial suspension was prepared by transferring three colonies into a tube containing 5 ml of normal saline. The turbidity of the suspension was adjusted to match the 0.5 McFarland standard. Using a sterile swab, the suspension was evenly distributed onto Mueller-Hinton agar. Antimicrobial disks were placed on the agar surface, and the plates were allowed to sit for 30 minutes to promote antibiotic diffusion before being incubated at 35 ± 2°C for 24 hours [18,19]. The antibiotics tested included Cefoxitin (30 mcg) for MRSA identification, vancomycin (30 µg), erythromycin (15 µg), clindamycin (2 µg), penicillin (10 units), gentamicin (10 µg), doxycycline (30 µg), ciprofloxacin (5 µg), cotrimoxazole (1.25/23.75 µg), and chloramphenicol (30 µg). A standard strain of *S. aureus* (ATCC 25923) was used as a quality control.

### Data analysis

The data were analyzed using SPSS version 20, employing descriptive statistics, frequencies, and percentages. A chi-square test was conducted to study the association between MRSA prevalence and categorical variables, including age, gender, education, and occupation. In addition, binary logistic regression was applied to identify the factors associated with MRSA prevalence. A confidence level of 95% (*P* <0.05) was established to assess statistical significance.

### Ethical consideration

Ethical approval for the verbal consent was granted from the Jamhuriya University Research Ethics Committee (reference number JUREC0050/FMHS00032/092023). Written consent was not obtained due to cultural preferences and potential literacy barriers. Instead, a trained research assistant read the consent form aloud to participants in Somali, and verbal agreement was documented in a research logbook, with a nurse serving as a witness.

## Results

### Profile of the study participants

The study included 418 participants, with 56.4% male and 43.6% female. The majority of participants were in the 21-30 years age group (46.0%), followed by the 31-40 years age group (27.0%). In terms of education, the participants had various education levels, with 27.0% having attended Madrasa, 38.6% having completed primary education, 25.1% having finished secondary education, and 9.2% having a university education. Regarding occupation, 24.9% were unemployed, 14.2% were farmers, 14.2% were students, 38.6% were housewives, and 8.1% were merchants. Furthermore, the majority of participants resided in urban areas (91.9%), whereas a smaller proportion lived in rural areas (8.1%), as shown in Table 1.

**Table 1:** Demographic features of study participants.

Variables	Frequency	Percent
**Sex**		
Male	238	56.4
Female	184	43.6
**Age**		
<20 years	56	13.3
21-30 years	194	46.0
31-40 years	114	27.0
40-50 years	52	12.3
>60 years	6	1.4
**Education**		
Madrasa	114	27.0
Primary	163	38.6
Secondary	106	25.1
University	39	9.2
**Occupation**		
Jobless	105	24.9
Farmer	60	14.2
Student	60	14.2
Housewife	163	38.6
Merchant	34	8.1
**Residence**		
Rural	34	8.1
Urban	388	91.9

### Prevalence and risk factors of methicillin-resistant S. aureus

The study found a 26.7% prevalence of MRSA in 31 of 116 isolates, with a higher proportion in males (24.9%), whereas a lower prevalence was noted among individuals under 20 years old (14.3%) as shown in Table 2. This may be attributed to reduced exposure to health care settings, less frequent use of antibiotics, and a generally stronger immune response. In addition, younger individuals often have fewer comorbidities, which may lower their risk of acquiring MRSA. MRSA was more common in patients with deep wounds than those with superficial wounds, as shown in Table 3. Participants with deep wounds had significantly higher odds of methicillin resistance than those with superficial wounds (adjusted odds ratio [AOR]: 4.32, 95% confidence interval [CI]: 1.042-17.92, *P* <0.044). Participants with a hospital stay longer than 72 hours also had significantly higher odds of methicillin resistance (AOR: 4.1, 95% CI: 1.22-13.80, *P* <0.022). In addition, participants with a previous history of antibiotic use had higher odds of methicillin resistance (AOR: 1.12, 95% CI: 0.17-7.15, *P* <0.018). However, we were unable to categorize the specific types of antibiotics used because detailed records were not consistently available, which limits further interpretation of this association. Other factors, such as site of infection, type of wound, diabetes, previous history of wound infection, and currently being on antibiotics, did not show significant associations with MRSA, as shown in Table 3.

**Table 2:** Association between sociodemographic characters and methicillin-resistant *Staphylococcus aureus*.

Variables	Category	Susceptibility of methicillin N (%)		*P*-value
		Sensitive N (%)	Resistant N (%)	
Sex	Male	58(73.4%)	21 (26.6%)	
	Female	27 (73.0%)	10 (27.0%)	0.564
Age	<20 years	12 (85.7%)	2 (14.3%)	
	21-30 years	40 (75.5%)	13 (24.5%)	
	31-40 years	18 (64.3%)	10 (35.7%)	0.366
	40-50 years	12 (66.7%)	6 (33.3%)	
	>60 years	3 (100)	0 (0.0%)	
Education	Madrasa	25 (73.5%)	9 (26.5%)	
	Primary	32(72.7%)	12 (27.3%)	
	Secondary	18 (69.2%)	8 (30.8%)	0.780
	University	10 (83.3%)	2 (16.7%)	
Occupation	Jobless	34(79.1%)	9 (20.9)	
	Farmer	15 (71.4%)	6 (28.6%)	
	Student	11 (68.8%)	5 (31.2%)	0.405
	Housewife	20 (66.7%)	10 (33.3%)	
	Merchant	5 (83.3%)	1 (16.7%)	
Residence	Rural	9 (69.2%)	4 (30.8%)	0.477
	Urban	76 (73.8%)	27 (26.2%)	
The overall prevalence of methicillin-resistant *S. aureus*			26.7%

**Table 3:** Factors associated with methicillin-resistant *Staphylococcus aureus*.

Variables	Susceptibility of methicillin		Crude odds ratio with 95% confidence interval	Adjusted odds ratio with 95% confidence interval	*P*-value
	Sensitive	Resistant			
**Site of the wound**					
Leg	26	8	0.462 (0.065-3.266)	0.743 (0.077-7.180)	
Foot	27	10	0.556 (0.081-3.83)	0.527 (0.059-4.629)	
Hand	15	7	0.700(0.095-5.18)	0.970 (0.098-9.567)	0.880
Head and neck	5	2	0.600(0.053-6.795)	1.478 (0.089-24.566)	
Back abdomen	10	2	0.300 (0.029-3.135)	0.413 (0.413-6.775)	
Others	3	2	1	1	
**Depth of the wound**					
Deep	51	26	3.640 (1.274-10.402)	4.322 (1.042-17.920)	0.044
Superficial	35	5	1	1	
**Nature of the wound**					
Post Surgical wound	40	13	0.71 (0.270-1.895)	0.864 (0.290-2.571)	
Nonhealing ulcer	7	3	0.943 (0.201-4.422)	0.857 (0.143-5.150)	
Burn wound	4	1	0.550 (0.054-5.570)	0.529 (0.044-6.296)	0.991
Trauma	13	4	0.677 (0.176-2.604)	0.9612 (0.190-4.873)	
Abscess	22	10	1	1	
**Diabetes**					
Yes	9	77	1.267 (0.361-4.454)	4.105 (1.221-3.528)	0.763
No	4	27	1	1	
**Hospital stay >72 hours**					
Yes	11	75	3.247 (1.214-8.682)	4.105 (1.221-13.8004)	0.022
No	10	21	1	1	
**Previous history of antibiotic**					
Yes	58	28	3.259 (1.039-10.218)	1.1224 (0.176-7.1576)	0.018
No	27	4	1	1	
**History of wound infection**					
Yes	40	46	1.396 (0.612-3.185)	5.343 (1.329-21.4701)	0.903
No	17	14	1	1	
**Currently on antibiotics**					
Yes	40	46	1.592 (0.695-3.651)	0.6003 (0.6003-4.170)	0.606
No	18	13	1	1	

### Antibiotic profile of methicillin-resistant S. aureus

All isolates were 100% resistant to penicillin, and all MRSA strains were 100% susceptible to vancomycin. MRSA showed resistance rates of 16.1% to clindamycin, 32.3% to doxycycline, 29% to chloramphenicol, and 61.3% to erythromycin, as shown in Table 4.

**Table 4:** Antibiotic susceptibility of methicillin-resistant *Staphylococcus aureus* in the study area.

Antibiotics	Disk concentration	Resistance pattern of methicillin-resistant *S. aureus* (N = 31)	
		Sensitive N (%)	Resistant N (%)
Penicillin	10 units	0 (0)	31 (100)
Ciprofloxacin	5 mcg	15 (48.4)	16 (51.6)
Cotrimoxazole	1.25/23.75 mcg	13 (42)	18 (58)
Doxycycline	30 g	21 (67.7)	10 (32.3)
Erythromycin	15 mcg	12 (38.7)	19 (61.3)
Clindamycin	2 mcg	26 (83.9)	5 (16.1)
Chloramphenicol	30 mcg	22 (71)	9 (29)
Gentamycin	10 mcg	14 (45.2)	17 (54.8)
Vancomycin	30 µg	31 (100)	0 (0)

## Discussion

MRSA is a critical worldwide health concern for its resistance to antibiotics, leading to difficult, costly, and, sometimes, untreatable infections, with increased health care costs and mortality [18]. The situation is worsened by limited resources, poor infection control, and antibiotic misuse [19]. In Somalia, the high prevalence of MRSA further strains an already fragile health care system, making targeted interventions and effective infection control essential in such under-resourced settings [14,20]. This study found an overall prevalence of MRSA (26.7%), which is similar to a previous study conducted in Ethiopia, higher than the prevalence reported in Kenya, and lower than another study carried out in Ethiopia [[21], [22], [23]]. Most MRSA cases are likely to be hospital-acquired due to the association with deep wounds and prolonged hospital stay. However, we lacked molecular typing to distinguish between community-acquired and nosocomial MRSA strains.

Our study found several risk factors that are significantly associated with MRSA. Deep wounds were 4.46 times more likely to be associated with MRSA, and this is similar to other studies that found that deep wounds tend to have higher resistance rates [23]. Most deep-wound MRSA cases were reported within the surgical ward, which may suggest localized transmission or a potential outbreak. However, due to the cross-sectional nature of this study, no formal outbreak investigation was conducted. Patients hospitalized for more than 72 hours were 9.20 times more likely to have methicillin-resistant MRSA. This supports previous research that longer hospital stays increase the risk of antibiotic resistance [24]. A history of antibiotic use increased the likelihood of methicillin resistance by 3.20 times; this is similar to other studies that indicate previous antibiotic exposure raises the risk of resistance [17,23], and it’s opposite to the study conducted in Tanzania [10].

The antibiotic susceptibility pattern of MRSA isolates reveals significant resistance to several commonly used antibiotics, such as penicillin (100%), presenting substantial treatment challenges. The high resistance of MRSA isolates to penicillin is due to β-lactamase production and the presence of the mecA gene, which reduces the efficacy of β-lactam antibiotics [25,26]. This finding aligns with some studies from Ethiopia [27]. Although penicillin is ineffective against MRSA due to resistance, vancomycin remains a key treatment, although its use can be limited by availability and the need for monitoring in resource-constrained areas, such as Somalia. All MRSA strains were sensitive to vancomycin is consistent with previous study [28] but does not eliminate the need for ongoing surveillance. Ciprofloxacin shows moderate effectiveness against MRSA, with nearly equal rates of sensitivity and resistance. This effectiveness is somewhat better than a previous study that reported a 61.5% resistance rate [27]). Fluoroquinolones, such as ciprofloxacin, are used in Somalia, but the lack of regulation and specific national MRSA treatment guidelines may lead to improper prescribing. The widespread availability of unregulated medicines and a lack of public awareness about appropriate antibiotic use are significant challenges in the country. Notably, studies have indicated a connection between the hospital use of fluoroquinolones and the rise of MRSA infections [29]. Cotrimoxazole showed limited efficacy, with 42% sensitivity and 58% resistance, comparable to the study by Tsige et al. [27], who found a 53.8% resistance rate. Doxycycline was more effective in our study, with 67.7% sensitivity and 32.3% resistance, supporting its use in MRSA treatment. This is consistent with the findings of Adhikari et al. [30], which reported a 71.2% sensitivity rate. Erythromycin demonstrated high resistance (61.3%) and low sensitivity (38.7%); this is higher than the previous study [31], which reported a 11% resistance rate. Clindamycin showed high sensitivity (83.9%) and low resistance (16.1%), making it a strong candidate for MRSA therapy; this is higher than the findings of Adhikari et al. [32], who observed a 95.5% sensitivity rate. Chloramphenicol was effective, with 71% sensitivity and 29% resistance, which is different from previous data showing an 89.3% sensitivity rate and 10.7% resistance [30]. Gentamycin had concerning resistance levels (54.8%) and lower sensitivity (45.2%), which is higher than the findings of Tsige et al. [27], who reported a (53.8%) resistance rate.

Although standard infection control measures, such as hand hygiene and wound dressing, are in place, the absence of formal antibiotic stewardship programs in Somali health care settings is a critical gap. These findings emphasize the urgent need to implement structured antimicrobial stewardship and robust infection control strategies across the nation. Such programs are essential for effective MRSA management, requiring ongoing surveillance of antibiotic resistance and treatment tailored to local susceptibility patterns.

## Conclusion

The study found a 26.7% prevalence of MRSA, with deep wounds, hospital stays longer than 72 hours, and previous antibiotic determined as significant risk factors. This underscores the need for effective infection control measures and antimicrobial stewardship.

## Limitations

The study is limited by its cross-sectional design, which could not establish causality, and its hospital-based setting, limiting applicability to other regions. We did not perform the D-zone test to assess inducible clindamycin resistance, which may have underestimated resistance rates. In addition, we were unable to categorize the specific antibiotics used before MRSA infection, and we lacked molecular confirmation of resistance genes.

## Author Contributions

All authors contributed equally to the conception and design of the study, data collection, laboratory analysis, data interpretation, manuscript drafting, and final approval of the version to be published.

## Declaration of competing interest

The authors have no competing interests to declare.
